# Supplementary material for: Systematically dissecting the global mechanism of miRNA functions in mouse pluripotent stem cells
Source: BMC Genomics. 2015 Jul 1;16(1):490. doi: 10.1186/s12864-015-1706-y (PMC4488055; doi:10.1186/s12864-015-1706-y)
Supplement: Additional file 1: — Figure S1. Functional enrichments of all targets that are targeted by all up- and down-regulated miRNAs. A, Up-regulated miRNAs. B, down-regulated miRNAs. Figure S2. Total 17 out of top 20 important miRNAs target developmental genes. Figure S3. Down-regulated miRNAs mediate metabolism in stem cells. Figure S4. All pluripotent genes targeted by up-regulated miRNAs (A) and down-regulated miRNAs (B) in stem cells. Figure S5. An activated network targeted by down-regulated miRNAs. Figure S6. Down-regulated miRNAs targeting DNMT3A. Table S1. Data sources. Table S2. A list of miRNAs that are frequently and significantly differential expressed between stem cells and somatic cells. Table S3. A list of genes coding for proteins that are frequently and significantly differential expressed between stem cells and somatic cells. Table S4. A list of top 20 important miRNAs. [file 12864_2015_1706_MOESM1_ESM.pdf]

Systematically Dissecting the Global Mechanism of MiRNA functions in Stem Cells

Anyou Wang ,Yan Zhong, Qianchuan He

Supplemental materials

Figure S1, Functional enrichments of all targets that are targeted by all up- and down-regulated miRNAs. A, Up-regulated miRNAs. B, down-regulated miRNAs.

Figure S2, total 17 out of top 20 important miRNAs target developmental genes.

Figure S3, down-regulated miRNAs mediate metabolism in stem cells.

Figure S4, all pluripotent genes targeted by up-regulated miRNAs (A) and down-regulated miRNAs (B) in stem cells.

Figure S5. An activated network targeted by down-regulated miRNAs.

Figure S6. Down-regulated miRNAs targeting DNMT3A.

Table S1, Data sources

Table S2, a list of miRNAs that are frequently and significantly differential expressed between stem cells and somatic cells.

Table S3, a list of genes coding for proteins that are frequently and significantly differential expressed between stem cells and somatic cells.

Table S4, a list of top 20 important miRNAs

Figure S1

A

Top 10 GO term based on entire annotation

p-value	corr p-valu	Description
3.62E-86	1.11E-82	cellular process
4.26E-65	6.52E-62	multicellular organismal development
1.59E-61	1.62E-58	anatomical structure development
2.27E-61	1.74E-58	developmental process
5.6E-61	3.42E-58	system development
8.15E-53	4.15E-50	anatomical structure morphogenesis
2.18E-48	9.55E-46	biological regulation
1.15E-47	4.38E-45	regulation of biological process
9.41E-47	3.2E-44	organ development
6.01E-44	1.84E-41	positive regulation of biological process

Top 10 GO term using reference of a set of genes altered expresion

p-value	corr p-valu	Description
1.43E-08	2.10E-05	anatomical structure morphogenesis
1.08E-07	7.95E-05	organ development
2.95E-07	1.45E-04	developmental process
5.06E-07	1.87E-04	anatomical structure development
6.41E-07	1.89E-04	multicellular organismal development
1.57E-06	3.85E-04	system development
3.54E-06	7.45E-04	anatomical structure formation involved in morphogenesis
1.36E-05	2.50E-03	multicellular organismal process
1.95E-05	3.20E-03	cell development
3.33E-05	4.91E-03	striated muscle tissue development

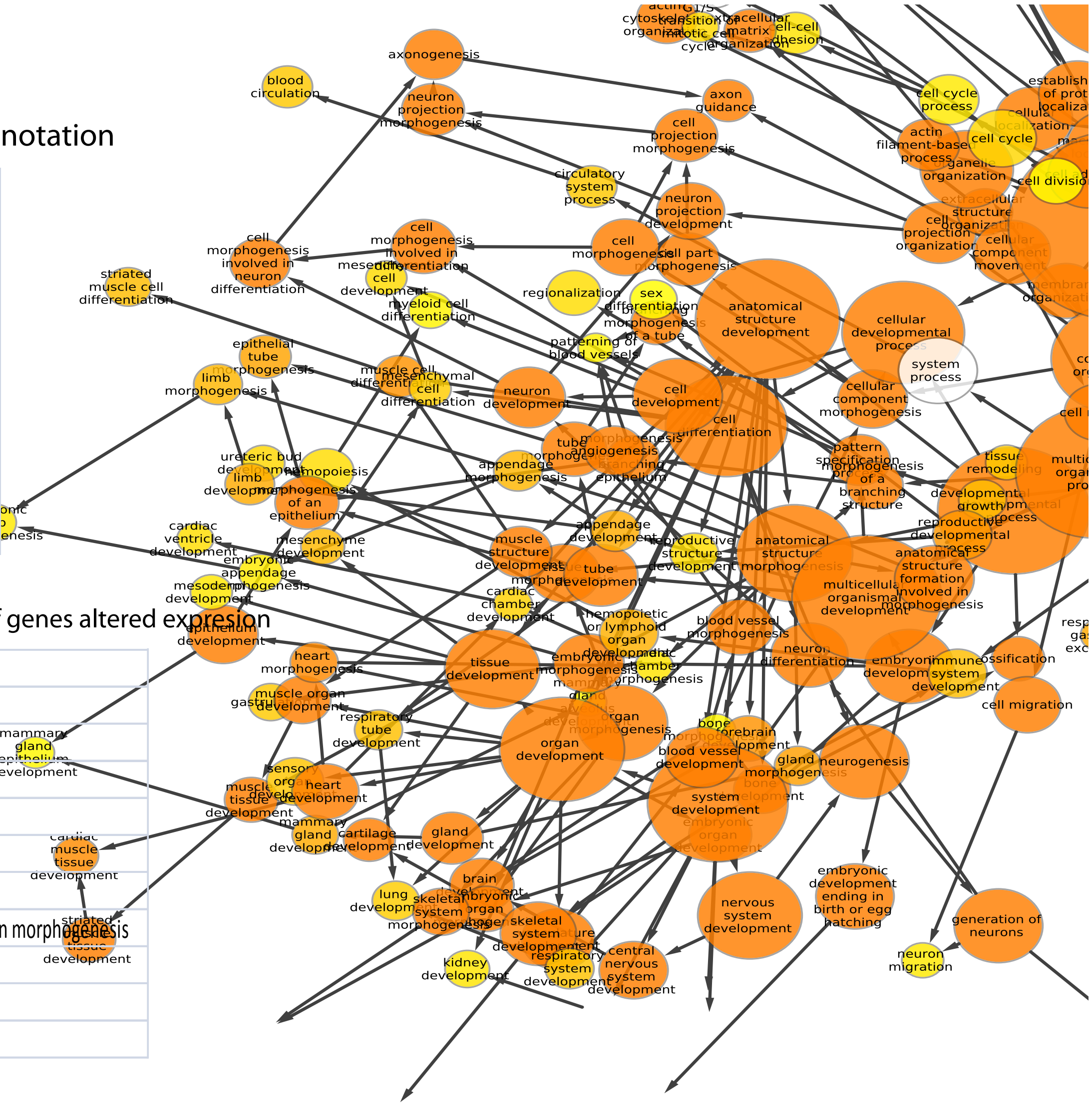

B

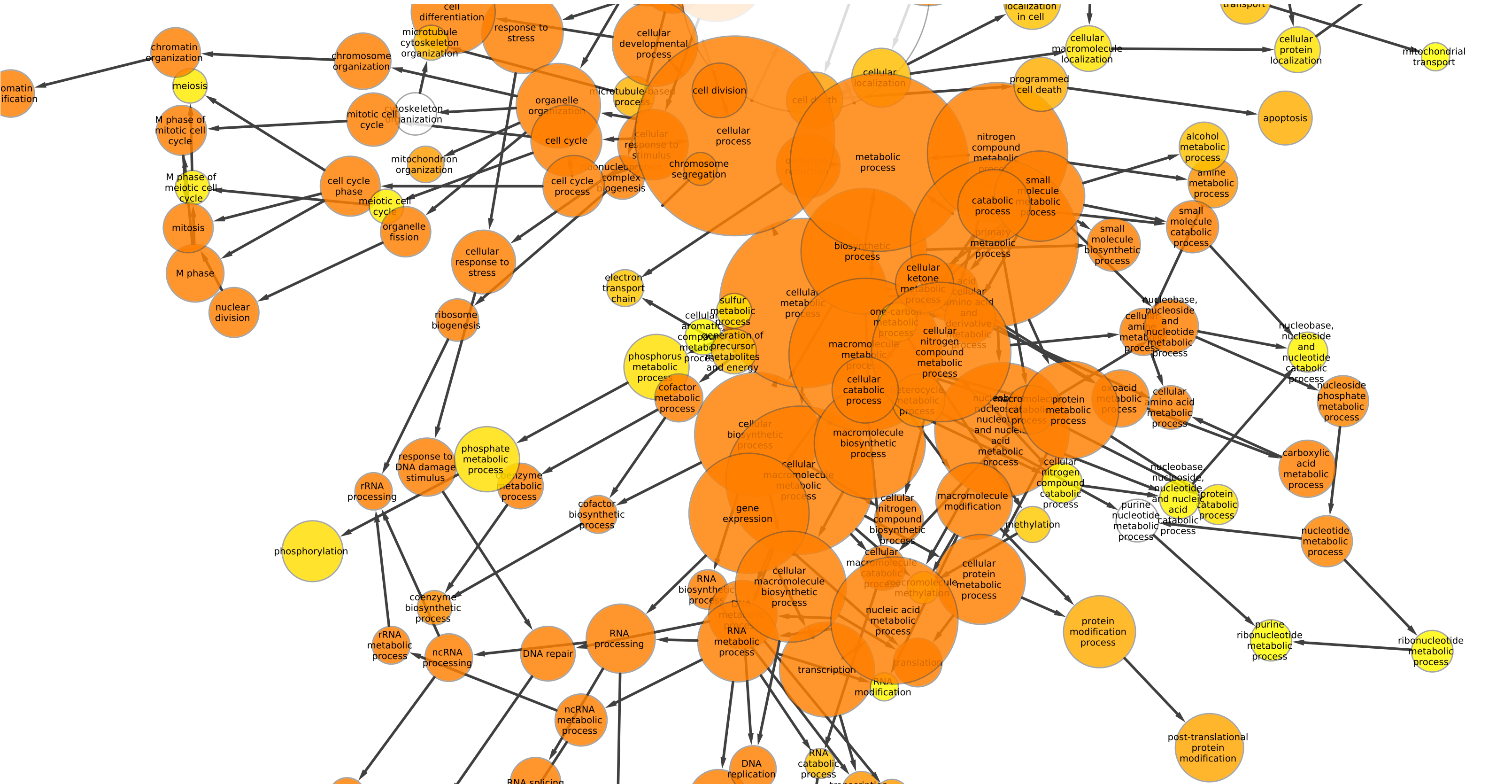

Total 17 out of top 20 miRNAs target developmental genes

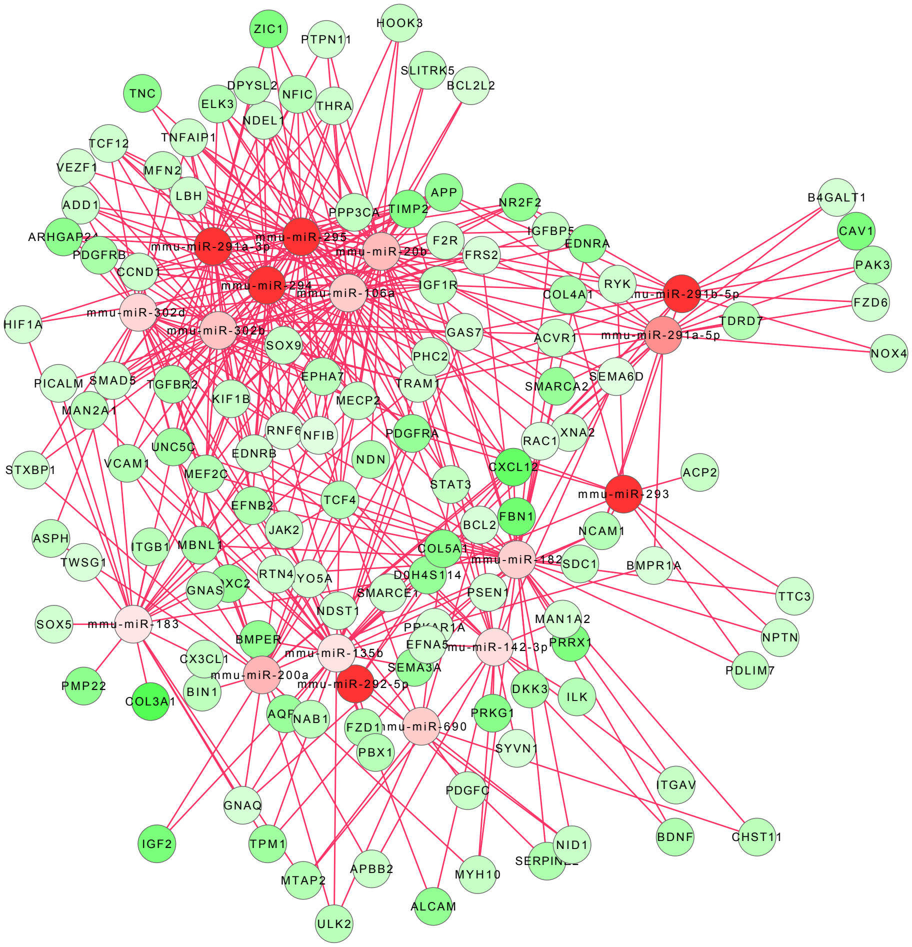

Down-regulated miRNAs mediate metabolism and pluripotency

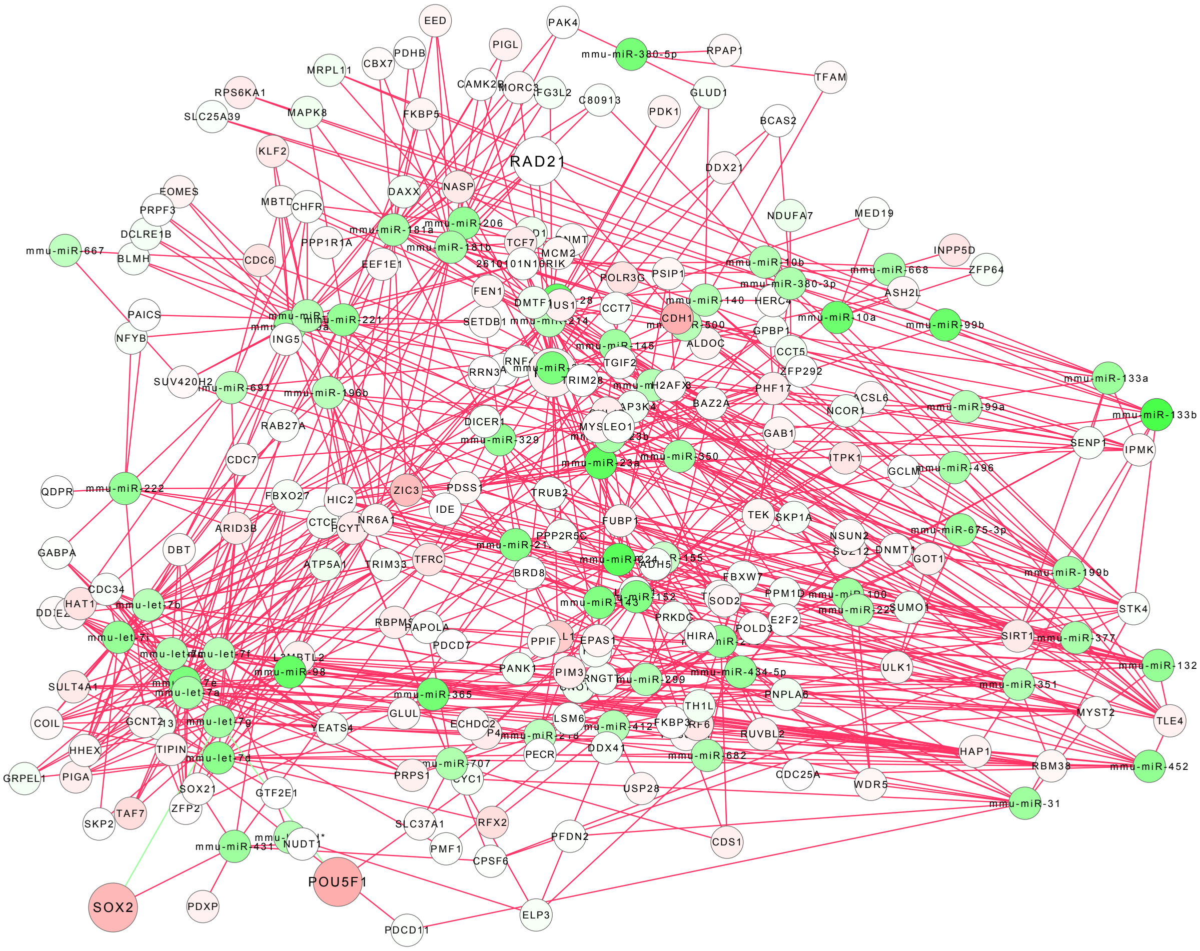

A

Pluripotent genes targeted by up-regulated miRNAs in stem cells

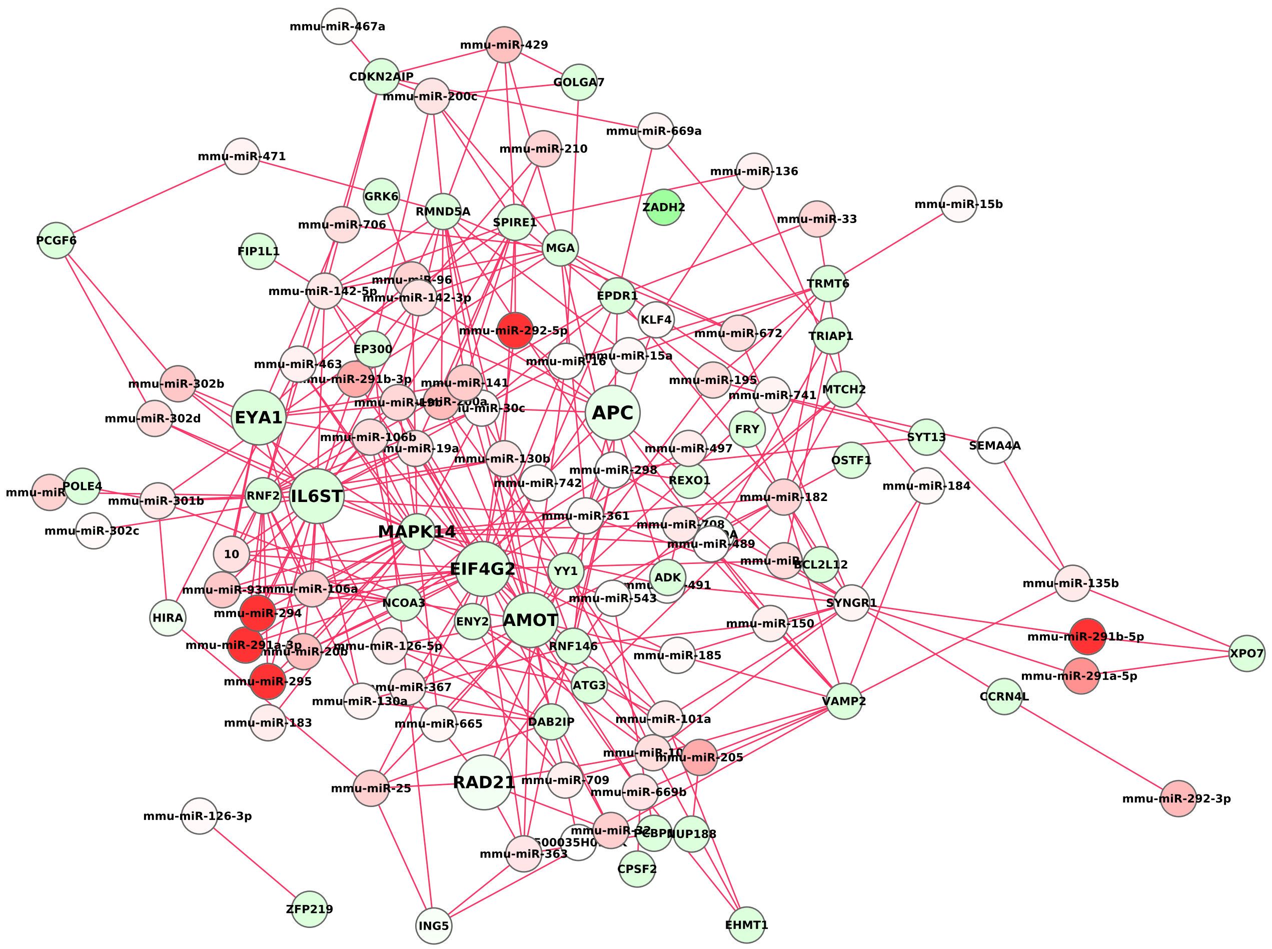

B

Pluripotent genes targeted by down-regulated miRNAs in stem cells

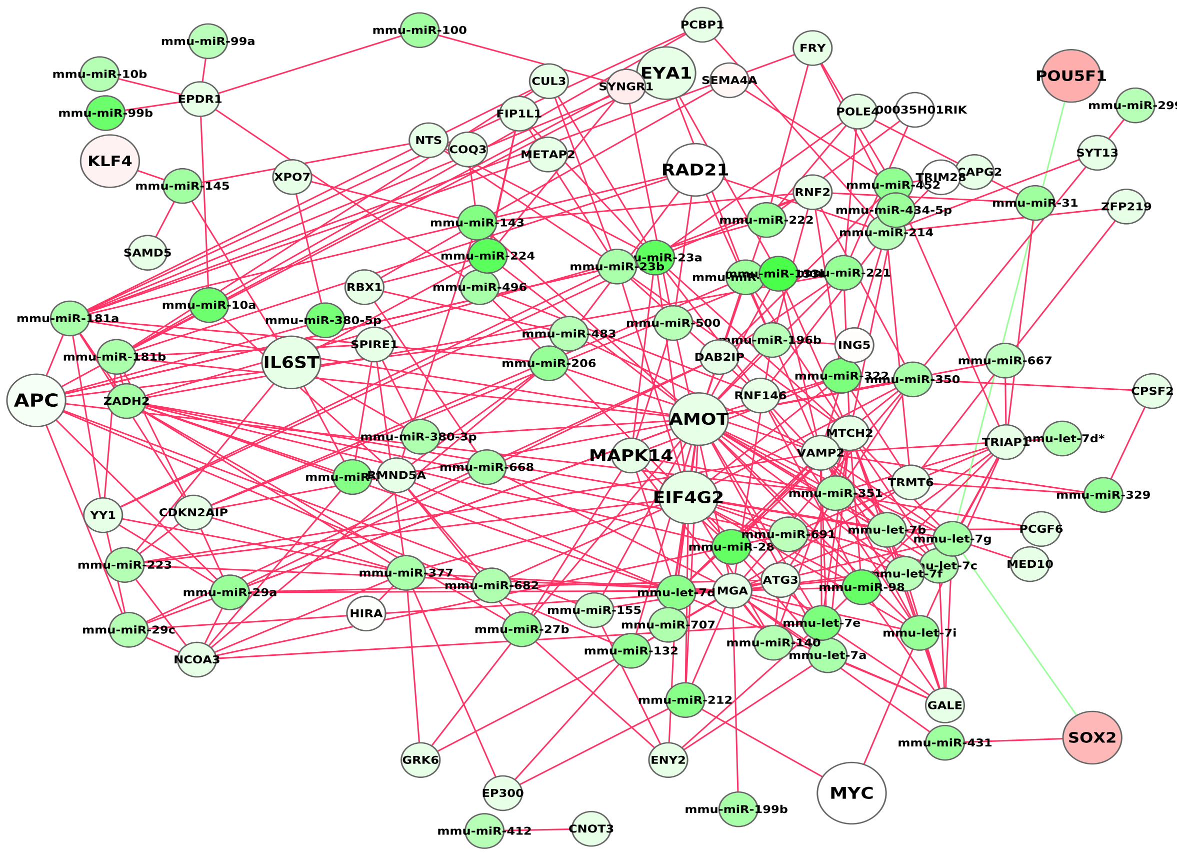

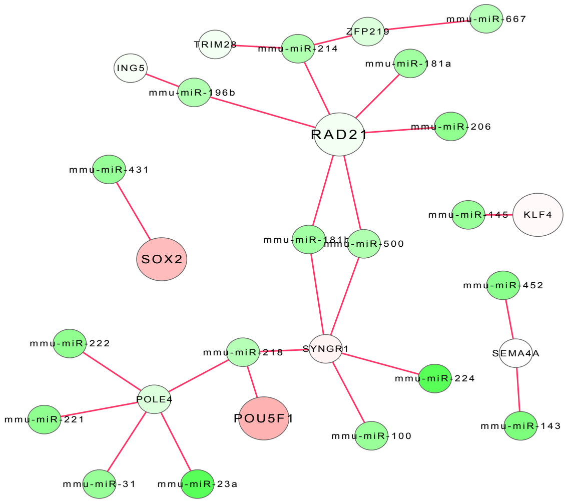

mmu-miR-667
TRIM28
mmu-miR-214
ZFP219
mmu-miR-181a
ING5
mmu-miR-196b
RAD21
mmu-miR-206
mmu-miR-431
mmu-miR-145
KLF4
mmu-miR-181b
mmu-miR-500
SOX2
mmu-miR-452
mmu-miR-222
mmu-miR-218
SYNGR1
SEMA4A
mmu-miR-224
POLE4
mmu-miR-221
POU5F1
mmu-miR-143
mmu-miR-100
mmu-miR-31
mmu-miR-23a

Figure S6

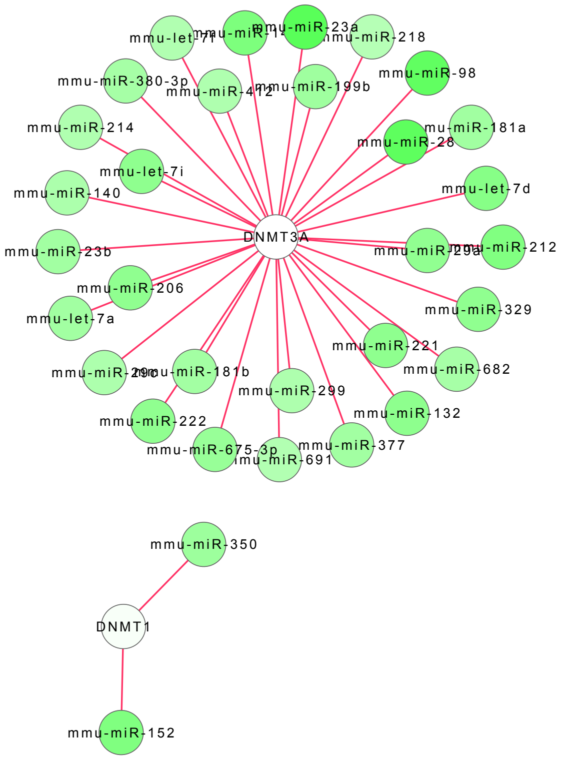
mmu-miR-23a
mmu-miR-218
mmu-miR-380-3p
mmu-miR-412
mmu-miR-199b
mmu-miR-98
mmu-miR-214
mmu-miR-28
mmu-let-7i
mmu-miR-140
mmu-let-7d
DNMT3A
mmu-miR-23b
mmu-miR-212
mmu-miR-206
mmu-miR-329
mmu-let-7a
mmu-miR-221
mmu-miR-682
mmu-miR-181b
mmu-miR-299
mmu-miR-132
mmu-miR-222
mmu-miR-377
mmu-miR-675-3p
mmu-miR-350
DNMT1
mmu-miR-152

Table S1 data resources

GEO number	Platform	measurement
GSE25310	CLIP-seq	ES miRNA targets
GSE11724	ChIP-seq	TF binding
GSE11431	ChIP-seq	TF binding
GSM278905	RRBS	ES DNA methylation
GSM278902	RRBS	TKO methylation
GSM539867	RNA-Seq	mouse embryonic fibroblast cells [09-002]
GSM539866	RNA-Seq	mouse embryonic stem cells [09-002]
GSE30012	Mouse430_2 array	ES miRNA expression
GSE25310	GPL11410	micrRNA and mRNA expression
GSM231739	Agilent-GPL2872	ES gene expression TKO/WT
GSE15267	AffymetrixGPL1261	Gene expression somatic cells vs stem cells
GSE14012	AffymetrixGPL1261	Gene expression somatic cells vs stem cells
GSE10871	AffymetrixGPL1261	Gene expression somatic cells vs stem cells
GSE14790	AffymetrixGPL1261	Gene expression somatic cells vs stem cells
GSE16062	IlluminaGPL6885	Gene expression somatic cells vs stem cells

Table S2 miRNAs differentially expressed between stem cell vs somatic cells with high frequency >25% of observations

geneID	foldChange.StemcellvsSomaticCells
mmu-miR-291a-3p	23.38772103
mmu-miR-292-5p	20.9773225
mmu-miR-295	18.61514609
mmu-miR-290	16.83638073
mmu-miR-291b-5p	16.74397919
mmu-miR-294	16.33979504
mmu-miR-293	15.71944751
mmu-miR-291a-5p	10.70302558
mmu-miR-291b-3p	8.775662583
mmu-miR-205	8.656636965
mmu-miR-292-3p	7.513040922
mmu-miR-200a	7.476828136
mmu-miR-20b	7.113430578
mmu-miR-429	6.970376253
mmu-miR-93	6.320621674
mmu-miR-302b	6.319533201
mmu-miR-124a	6.210366092
mmu-miR-141	6.112532011
mmu-miR-32	5.892597823
mmu-miR-25	5.664093286
mmu-miR-106a	5.660199796
mmu-miR-96	5.576097803
mmu-miR-690	5.566673137
mmu-miR-210	5.494180249
mmu-miR-182	5.380304319
mmu-miR-339	5.178218031
mmu-miR-33	5.150748639
mmu-miR-19b	5.130661066
mmu-miR-302d	4.786597786
mmu-miR-195	4.773097795
mmu-miR-297b	4.742119218
mmu-miR-499	4.699375408
mmu-miR-106b	4.67924418
mmu-miR-712*	4.616388282
mmu-miR-101b	4.615452785
mmu-miR-706	4.577798951
mmu-miR-672	4.425689091
mmu-miR-20a	4.355849211
mmu-miR-302	4.187271105
mmu-miR-142-3p	4.185529766
mmu-miR-19a	4.154314537
mmu-miR-450b	4.143615059
mmu-miR-200c	4.139625599
mmu-miR-17-5p	4.015734124

mmu-miR-669b	4.005643461
mmu-miR-363	4.004376577
mmu-miR-130b	3.921190227
mmu-miR-142-5p	3.771559152
mmu-miR-708	3.757978939
mmu-miR-301b	3.671137996
mmu-miR-297	3.649760196
mmu-miR-711	3.642487186
mmu-miR-135b	3.61287415
mmu-miR-145	-3.689012325
mmu-miR-125b	-3.750522829
mmu-miR-675-3p	-3.775797104
mmu-miR-31	-3.789955248
mmu-miR-431	-3.827836369
mmu-miR-133a	-3.931634792
mmu-miR-434-5p	-3.95970537
mmu-miR-29a	-4.128390595
mmu-miR-125a	-4.168574915
mmu-miR-27b	-4.181204463
mmu-miR-206	-4.1991836
mmu-miR-222	-4.206742949
mmu-miR-329	-4.226853284
mmu-let-7i	-4.253606445
mmu-miR-221	-4.277690509
mmu-miR-132	-4.327457901
mmu-miR-345	-4.362726416
mmu-miR-452	-4.493999108
mmu-let-7d	-4.733310501
mmu-miR-181a*	-4.890038681
mmu-miR-212	-5.03014549
mmu-miR-152	-5.091913825
mmu-miR-143	-5.251965264
mmu-miR-433-5p	-5.353386842
mmu-let-7e	-5.627257306
mmu-miR-322	-5.627467759
mmu-miR-380-5p	-5.840198038
mmu-miR-666	-5.963478682
mmu-miR-365	-6.053925857
mmu-miR-10a	-6.463178387
mmu-miR-24*	-6.501407687
mmu-miR-99b	-6.54057846
mmu-miR-98	-6.824098585
mmu-miR-28	-6.985496746
mmu-miR-23a	-7.258185943
mmu-miR-675-5p	-7.319121705
mmu-miR-224	-7.383165325
mmu-miR-133b	-8.331863729

mmu-miR-155	-12.36089174

Table S3. Genes differentially expressed between stem cells and somatic cells with high frequency >50% of observations

GB_ACC	ENTREZ_GI	Gene.Symbol	logFoldChange.ESvsSC
BC006640	20315	Cxcl12	-7.932773867
BF225802	16011	Igfbp5	-7.7652649
AW550625	12825	Col3a1	-7.7405454
BF227507	12843	Col1a2	-7.50996995
BB542051	18295	Ogn	-7.13772715
U08020	12842	Col1a1	-7.025342217
NM_013655	20315	Cxcl12	-6.8464668
NM_011581	21826	Thbs2	-6.743730283
AV229424	12832	Col5a2	-6.6650371
BB051738	18933	Prrx1	-6.523803517
M65143	16948	Lox	-6.263793183
AF007248	14118	Fbn1	-6.234405867
NM_009933	12833	Col6a1	-6.142877467
NM_007993	14118	Fbn1	-6.087729233
BC019502	12111	Bgn	-6.042548083
AB015978	18414	Osmr	-6.018220117
BE197945	20324	Sdpr	-5.902171217
NM_007729	12814	Col11a1	-5.888984717
AW049660	18032	Nfix	-5.878165733
NM_010514	16002	Igf2	-5.805814817
BB197591	54216	Pcdh7	-5.793524283
AV226618	13837	Epha3	-5.703367667
AF378762	69538	Antxr1	-5.671588117
M68513	13837	Epha3	-5.646988233
BC002064	19242	Ptn	-5.631718783
NM_011340	20317	Serpinf1	-5.561155483
BB795075	18214	Ddr2	-5.5521091
BC023060	216616	Efemp1	-5.541377517
BF144658	21808	Tgfb2	-5.516599417
BB250384	22329	Vcam1	-5.511377133
AB029929	12389	Cav1	-5.478985417
D67076	11504	Adamts1	-5.469247617
BB315728	18032	Nfix	-5.460392167
BI794771	12842	Col1a1	-5.456734267
L06502	18933	Prrx1	-5.448142767
BG793483	21813	Tgfbr2	-5.434682533
BB532202	14264	Fmod	-5.394454433
BB361162	22771	Zic1	-5.383117233
AI931862	12111	Bgn	-5.378983617
BC014690	21809	Tgfb3	-5.302384367
NM_018865	22402	Wisp1	-5.29682725
AV064339	20324	Sdpr	-5.2953667
BG067986	54216	Pcdh7	-5.295023283
NM_030888	81799	C1qtnf3	-5.279631533

BB233297	21826	Thbs2	-5.26170495
BF168458	21858	Timp2	-5.239630733
BB259670	14362	Fzd1	-5.23704885
AA499047	18596	Pdgfrb	-5.226616983
BC026446	219151	Scara3	-5.1983228
NM_010714	16876	Lhx9	-5.18046395
NM_133859	99543	Olfml3	-5.167943083
BB067079	22418	Wnt5a	-5.160633383
AW555664	399558	Flrt2	-5.14692565
BC021484	16651	Sspn	-5.141520367
BC011063	15402	Hoxa5	-5.109899467
M93954	21858	Timp2	-5.0922978
AV310588	12444	Ccnd2	-5.076240083
NM_013589	16997	Ltbp2	-5.07117345
BB791906	14178	Fgf7	-5.0607749
BQ175880	12444	Ccnd2	-5.031714083
NM_009829	12444	Ccnd2	-5.03108855
BC022107	12558	Cdh2	-5.030543317
NM_011160	19091	Prkg1	-5.010924967
AK007904	12444	Ccnd2	-5.010597567
BB114398	12816	Col12a1	-5.001727783
BB377340	13612	Edil3	-4.984431667
BI455189	12834	Col6a2	-4.981937467
BC018425	22418	Wnt5a	-4.949481167
AF309564	80837	Rhoj	-4.942552217
AW744319	12831	Col5a1	-4.912884333
NM_010284	14600	Ghr	-4.907539267
AK003674	68588	Cthrc1	-4.9040982
NM_009610	11468	Actg2	-4.88703015
BM220945	239217	Kctd12	-4.885967567
BB658835	23794	Adamts5	-4.884421383
BC019836	16010	Igfbp4	-4.86110935
NM_018764	54216	Pcdh7	-4.826503217
AA500897	26362	Axl	-4.824049367
AV222756	259300	Ehd2	-4.811690783
NM_018884	55983	Pdzrn3	-4.770790783
BB759833	17300	Foxc1	-4.766068317
BQ177170	17112	Tm4sf1	-4.749287983
BM250666	12830	Col4a5	-4.738634083
BC025502	231532	Arhgap24	-4.7154329
NM_007392	11475	Acta2	-4.71167825
NM_008885	18858	Pmp22	-4.71162355
AV246911	12831	Col5a1	-4.70608125
NM_133918	100952	Emilin1	-4.685038
AV260198	83675	Bicc1	-4.676352617
BC021376	225207	Zfp521	-4.6747989
NM_011607	21923	Tnc	-4.6736706

NM_007833	13179	Dcn	-4.668888083
AF357006	16949	Loxl1	-4.661639267
BC011507	74480	Samd4	-4.6606821
BB493031	23888	Gpc6	-4.659793267
BI249259	18032	Nfix	-4.659746767
NM_054044	78560	Gpr124	-4.652361
BC001999	13732	Emp3	-4.648468717
BB041180	16876	Lhx9	-4.631340583
BI690209	57342	Parva	-4.603038383
AK018128	70717	6330406I15Ri	-4.599817333
AI595932	17260	Mef2c	-4.571089667
AI463873	11819	Nr2f2	-4.558937633
BG075699	399558	Flrt2	-4.550595967
AV149705	108927	Lhfp	-4.537199083
AK014221	73230	Bmper	-4.536276183
BB468025	54381	Pgcp	-4.535907333
BB363812	14051	Eya4	-4.52926855
AK020118	23888	Gpc6	-4.5247486
BM217996	83675	Bicc1	-4.521218667
NM_009636	11568	Aebp1	-4.509546617
AA987181	15405	Hoxa9	-4.496607467
NM_011766	22762	Zfpm2	-4.496262983
AV315205	11658	Alcam	-4.495887367
U95030	11658	Alcam	-4.4742135
BF147716	17390	Mmp2	-4.463715417
NM_008608	17387	Mmp14	-4.4584154
AV332957	20429	Shox2	-4.44107
AK008112	18933	Prrx1	-4.429082483
BB787243	16010	Igfbp4	-4.408829283
M27130	12505	Cd44	-4.408205433
NM_023118	13132	Dab2	-4.401669267
BB817332	399558	Flrt2	-4.401003567
D63423	11747	Anxa5	-4.3868292
BC014870	235587	Parp3	-4.368841483
U03425	13649	Egfr	-4.353990467
NM_011150	19039	Lgals3bp	-4.34716645
BB229377	13717	Eln	-4.3469066
BF681826	26360	Angptl2	-4.34243245
AV369812	13649	Egfr	-4.340586717
AI385532	21825	Thbs1	-4.3388455
AK012411	20317	Serpinf1	-4.334218667
NM_016900	12390	Cav2	-4.327267733
NM_007471	11820	App	-4.32309575
BC026153	13841	Epha7	-4.322886683
BE652876	60527	Fads3	-4.31762715
NM_022563	18214	Ddr2	-4.317137833
BC005490	11820	App	-4.311839833

AY083458	235505	Cd109	-4.310650267
AW146109	12505	Cd44	-4.309742383
AF022889	268977	Ltbp1	-4.293317933
BF780807	23972	Papss2	-4.2837158
NM_010222	14231	Fkbp7	-4.283483917
BB765827	74761	Mxra8	-4.28266945
BB369191	27528	D0H4S114	-4.258134683
NM_011658	22160	Twist1	-4.252172233
BC022679	52552	Parp8	-4.249244683
BC024375	14600	Ghr	-4.235411317
AW558570	13617	Ednra	-4.235029783
NM_010151	13865	Nr2f1	-4.23266835
BB053506	207181	Rbms3	-4.227119367
BB464523	12293	Cacna2d1	-4.2176231
AI325255	14230	Fkbp10	-4.2139772
NM_009365	21804	Tgfb1i1	-4.2037574
AW537708	18595	Pdgfra	-4.201673433
BG244279	26360	Angptl2	-4.19122825
AV021105	208647	Creb3l2	-4.189322833
NM_021474	58859	Efemp2	-4.172999333
AW558468	230103	Npr2	-4.170204383
BM230959	229731	Slc25a24	-4.15558595
NM_013519	14234	Foxc2	-4.150217817
BG248060	12153	Bmp1	-4.1456309
AK011935	67155	Smarca2	-4.1424534
BC009660	109042	Prkcdbp	-4.138494033
NM_010730	16952	Anxa1	-4.137948167
NM_017464	18003	Nedd9	-4.125438817
NM_020606	57342	Parva	-4.117250083
NM_054042	70445	Cd248	-4.1162995
AW107196	74480	Samd4	-4.089650683
NM_008520	16998	Ltbp3	-4.088398567
NM_007400	11489	Adam12	-4.087465183
BB138485	70676	Gulp1	-4.06900915
BB041237	17389	Mmp16	-4.0669223
NM_009152	20346	Sema3a	-4.06663545
AK007400	77889	Lbh	-4.065124233
BC027199	233328	Lrrk1	-4.0638856
BC003726	14789	Leprel2	-4.061919033
BM220576	53623	Gria3	-4.059143167
BM570006	20564	Slit3	-4.050010933
NM_007472	11826	Aqp1	-4.049342233
BB329489	12406	Serpinh1	-4.035949167
BC024358	22004	Tpm2	-4.027799067
BB371406	57265	Fzd2	-4.024201683
BI106777	12293	Cacna2d1	-4.016322467
BB484759	224024	Scarf2	-3.995337517

BC025145	19266	Ptprd	-3.96890555
AK004519	56726	Sh3bgrl	-3.915095233
NM_010221	14230	Fkbp10	-3.911402667
BB114067	21345	Tagln	-3.906768233
NM_010517	16010	Igfbp4	-3.895716167
AV164956	22418	Wnt5a	-3.889125017
U71189	13592	Ebf2	-3.883624733
AF339910	18605	Enpp1	-3.88293505
BQ173967	13640	Efna5	-3.882553467
AV246882	235505	Cd109	-3.87793775
AK003186	22004	Tpm2	-3.875057833
NM_009821	12394	Runx1	-3.8666507
NM_010743	17082	Il1rl1	-3.865813883
BB324823	16998	Ltbp3	-3.86059405
BC025514	102644	Oaf	-3.857056783
NM_007801	13036	Ctsh	-3.85513275
NM_010581	16423	Cd47	-3.854950117
AV238225	16905	Lmna	-3.8510058
AK018679	16423	Cd47	-3.832084267
BG073383	15410	Hoxb3	-3.820933467
NM_026405	67844	Rab32	-3.819808667
BF144687	70676	Gulp1	-3.81964405
NM_019391	16985	Lsp1	-3.81878645
NM_013586	16950	Loxl3	-3.813466317
AI415741	94352	Loxl2	-3.8120326
BI220012	12406	Serpinh1	-3.811311317
NM_007802	13038	Ctsk	-3.804638867
BB832504	18028	Nfib	-3.801083117
BC013463	15430	Hoxd10	-3.784799
M22479	22003	Tpm1	-3.783677167
BG963150	20563	Slit2	-3.783541217
BC013560	12827	Col4a2	-3.774358167
NM_008984	19274	Ptprm	-3.769132717
BB233088	21928	Tnfaip2	-3.7684148
BC008277	13617	Ednra	-3.767968717
BI111620	21859	Timp3	-3.76779495
BB475194	23794	Adamts5	-3.76137165
NM_021355	14264	Fmod	-3.736082733
NM_007585	12306	Anxa2	-3.731015333
BF578055	229731	Slc25a24	-3.725295933
BB547877	20563	Slit2	-3.716729817
NM_016753	17035	Lxn	-3.70538055
AV016275	20742	Spnb2	-3.701173833
NM_008495	16852	Lgals1	-3.6991015
AW551930	83675	Bicc1	-3.698414167
AK018466	207181	Rbms3	-3.68959625
NM_008987	19288	Ptx3	-3.687359367

AK003819	74777	Sepn1	-3.6822946
AW412729	12816	Col12a1	-3.663389833
BC020152	223272	Itgbl1	-3.657189217
Y07687	18028	Nfib	-3.657044833
NM_011985	26561	Mmp23	-3.644890267
NM_009378	21824	Thbd	-3.6371471
BF451748	107589	Mylk	-3.626035767
BM251152	13003	Vcan	-3.620597833
BB468082	18481	Pak3	-3.6195631
NM_007899	13601	Ecm1	-3.6077534
BB522674	20541	Slc8a1	-3.606644633
NM_019989	56726	Sh3bgrl	-3.60465905
BC023448	23888	Gpc6	-3.603901967
AK002516	11857	Arhgdib	-3.6024453
BF100813	13618	Ednrb	-3.594391017
NM_011777	22793	Zyx	-3.594158817
BB326929	58194	Sh3kbp1	-3.582396833
BB248904	56726	Sh3bgrl	-3.58152235
BB027759	231997	Fkbp14	-3.5728165
AU021035	15529	Sdc2	-3.570693117
AI481026	29817	Igfbp7	-3.56210855
NM_008393	16373	Irx3	-3.560717517
BB526042	320452	P4ha3	-3.5487859
BB100920	17118	Marcks	-3.53907495
BB097480	223254	Farp1	-3.534434933
BI687652	18028	Nfib	-3.524464033
BG868949	17122	Mxd4	-3.514140767
NM_020561	57319	Smpdl3a	-3.51306225
AF059567	12579	Cdkn2b	-3.5073857
NM_009472	22253	Unc5c	-3.505214767
NM_009794	12334	Capn2	-3.498667433
NM_009627	11535	Adm	-3.48734415
BQ175796	18481	Pak3	-3.4733871
BC005569	58809	Rnase4	-3.460646467
BB327018	14118	Fbn1	-3.456580617
NM_010212	14200	Fhl2	-3.453920533
BB313689	20541	Slc8a1	-3.452686383
AK019164	17475	Mpdz	-3.437966567
AW702161	11745	Anxa3	-3.4355384
BC027084	259300	Ehd2	-3.43459445
BC005686	13713	Elk3	-3.43373535
BB065799	240725	Sulf1	-3.429931133
NM_130449	140792	Colec12	-3.424621383
BB823350	19092	Prkg2	-3.412943967
AI642438	16852	Lgals1	-3.4127125
NM_007855	13345	Twist2	-3.412527233
BB787292	14302	Frk	-3.411315533

BB354684	217410	Trib2	-3.410151567
AF117951	94352	Loxl2	-3.400751667
BC025897	225791	Zadh2	-3.398803383
NM_019877	56358	Copz2	-3.386908433
AV162270	22240	Dpysl3	-3.3799255
D63383	20465	Sim2	-3.378190883
NM_008546	17150	Mfap2	-3.375675317
NM_008008	14178	Fgf7	-3.373739283
BQ174721	214791	Sertad4	-3.37195165
BB377873	121021	Cspg4	-3.368490867
BB542535	13036	Ctsh	-3.3666448
BC024876	223453	Dap	-3.364419667
L24755	12153	Bmp1	-3.360524783
X66083	12505	Cd44	-3.354735533
AW547821	17268	Meis1	-3.3441435
BC016447	26427	Creb3l1	-3.342256433
BM213516	20742	Spnb2	-3.33549655
BM220880	108655	Foxp1	-3.326646233
AK004853	50781	Dkk3	-3.314331467
NM_008809	18596	Pdgfrb	-3.31185265
AV245241	259300	Ehd2	-3.304100233
NM_020007	56758	Mbnl1	-3.289487817
BC028307	68792	Srpx2	-3.28413355
AA709993	20365	Serf1	-3.275465433
BC006737	12370	Casp8	-3.272708067
BB151715	23871	Ets1	-3.271100167
BI452727	14314	Fstl1	-3.26508275
BB003393	21923	Tnc	-3.2549266
BB535494	18003	Nedd9	-3.2516385
BB779859	209448	Hoxc10	-3.2440814
BI248947	109624	Cald1	-3.241609283
L26349	21937	Tnfrsf1a	-3.2395265
BC012674	19285	Ptrf	-3.228721367
U30244	13642	Efnb2	-3.225253617
AK004598	71703	Armcx3	-3.218577033
BB075247	19266	Ptprd	-3.217064717
BB766878	107581	Col16a1	-3.216479983
NM_019586	56228	Ube2j1	-3.216275217
NM_008761	18301	Fxyd5	-3.21540255
M24849	22352	Vim	-3.212612833
BB366804	18230	Nxn	-3.208821517
AV031691	22771	Zic1	-3.207325183
AK003303	19266	Ptprd	-3.1900006
NM_009252	20716	Serpina3n	-3.1897822
BM233698	12977	Csf1	-3.186553767
AK018666	50766	Crim1	-3.184091083
NM_013813	13823	Epb4.1l3	-3.17559455

BB097063	16826	Ldb2	-3.167842717
BG070361	18514	Pbx1	-3.16060585
NM_023564	70310	Plscr3	-3.156913317
BC016893	15414	Hoxb6	-3.149439067
BC025602	52377	Rcn3	-3.143586083
BE628275	68910	Zfp467	-3.129177833
BB751459	240725	Sulf1	-3.118822083
NM_023813	108058	Camk2d	-3.106603767
BE630020	19122	Prnp	-3.099244017
NM_015786	50708	Hist1h1c	-3.096520833
NM_010736	17000	Ltbr	-3.096244717
NM_008380	16323	Inhba	-3.090835667
AF080090	20350	Sema3f	-3.086323
AV220340	229534	Pbxip1	-3.08320105
NM_016846	19731	Rgl1	-3.083172067
AA510713	56468	Socs5	-3.08271985
AV246759	21808	Tgfb2	-3.078846817
AV238718	233733	Galntl4	-3.07739925
BB311061	56876	Nelf	-3.07710745
BM231794	216188	Aldh1l2	-3.071789317
NM_053147	93893	Pcdhb22	-3.06913275
AV124445	17984	Ndn	-3.067003683
AI747133	12338	Capn6	-3.061495833
NM_009784	12293	Cacna2d1	-3.0553154
NM_008675	17965	Nbl1	-3.054173417
X58380	15364	Hmga2	-3.053552917
NM_009468	22240	Dpysl3	-3.0489959
NM_019390	16905	Lmna	-3.043480833
BB533903	50708	Hist1h1c	-3.028666667
BB250811	18542	Pcolce	-3.026433
BE951265	66180	1110036O03R	-3.02614895
AW546141	17118	Marcks	-3.021924833
BB825801	13713	Elk3	-3.021571583
NM_009255	20720	Serpine2	-3.02130325
BF158638	12826	Col4a1	-3.010500317
NM_016886	53623	Gria3	-3.003217433
NM_009925	12813	Col10a1	-3.001262167
AV278675	244448	Triml1	3.01398255
BG966751	104156	Etv5	3.0156244
X67668	97165	Hmgb2	3.020431667
BB089717	12449	Ccnf	3.022975367
BB292220	58180	Hic2	3.025155533
BG069191	50927	Nasp	3.027539567
BG066754	67204	Eif2s2	3.0295477
AK019115	68239	Krt42	3.03983065
AU015121	268697	Ccnb1	3.042294933
NM_008566	17218	Mcm5	3.058338333

NM_019812	93759	Sirt1	3.064211417
NM_020599	19771	Rlbp1	3.06822615
AV280841	219132	D14Ertd668e	3.078624667
BB493242	50927	Nasp	3.081964833
NM_022409	63872	Zfp296	3.082454667
NM_013873	29859	Sult4a1	3.0839579
AF302127	72388	Ripk4	3.086288367
NM_011597	21873	Tjp2	3.087218833
BF578266	227327	B3gnt7	3.0910465
AI195532	216456	Gls2	3.091450267
BE650359	13619	Phc1	3.103443667
BM247863	56380	Arid3b	3.10414725
BM935811	16403	Itga6	3.109543333
BG071670	75796	Cdyl2	3.110318667
BC006704	226419	Dyrk3	3.114882817
NM_008628	17685	Msh2	3.118807583
BM196098	14562	Gdf3	3.129241967
NM_020567	57441	Gmnn	3.129819017
AK011289	66953	Cdca7	3.133227817
NM_009426	22044	Trh	3.133924383
BB367422	239759	Liph	3.1340973
BC011474	16818	Lck	3.140572717
NM_011176	19143	St14	3.14224885
BB667216	22371	Vwf	3.143579333
BI739053	12725	Clcn3	3.159571817
NM_011487	20849	Stat4	3.169867333
BQ177140	218038	Amph	3.17188915
NM_008452	16598	Klf2	3.1728325
AF329833	66790	Grtp1	3.203848083
BB540053	217653	C79407	3.221807417
NM_007691	12649	Chek1	3.229280333
BC016095	228421	Kif18a	3.231334583
BC017621	223775	Pim3	3.23674225
U42190	17688	Msh6	3.242437417
NM_027288	110173	Manba	3.25011815
BE951628	217653	C79407	3.269844583
BB125424	242894	Actr3b	3.270038817
AK010648	70024	Mcm10	3.288071933
AK006509	75796	Cdyl2	3.304674333
NM_010174	14077	Fabp3	3.309141333
NM_053271	116838	Rims2	3.314384433
BG070553	110957	D1Pas1	3.318172517
NM_133664	16763	Lad1	3.323882083
BQ176661	54484	Mkrn1	3.3289175
AF237702	20425	Shmt1	3.331763533
NM_016851	54139	Irf6	3.333859083
AA543734	21386	Tbx3	3.333990817

NM_010346	14786	Grb7	3.34545575
BC016571	50799	Slc25a13	3.36053825
AK007201	14536	Nr6a1	3.360795183
NM_013514	13829	Epb4.9	3.361635267
U39203	16331	Inpp5d	3.380223367
AW552407	217837	Itpk1	3.380479
AK011462	72148	2610019F03R	3.381959433
BB397151	332221	Zscan10	3.400120667
U31625	12189	Brca1	3.4060535
BM225706	243574	Kbtbd8	3.415306283
NM_031386	83560	Tex14	3.416736917
NM_009171	20425	Shmt1	3.449195983
AK011986	112422	2610305D13R	3.475925817
AV305746	231327	Ppat	3.479371517
BI465857	12224	Klf5	3.483464667
NM_021308	57746	Piwil2	3.4897425
BB758095	21873	Tjp2	3.499096167
NM_016964	50878	Stag3	3.503349717
BG066919	17765	Mtf2	3.526635767
BE986504	71801	Plekhf2	3.556072867
AV277242	243963	Zfp473	3.576478483
NM_011799	23834	Cdc6	3.57881495
NM_026190	67486	Polr3g	3.6006572
NM_010357	14860	Gsta4	3.6286395
AU016127	67374	Jam2	3.629554333
BM230524	217944	Rapgef5	3.6361713
NM_022024	63986	Gmfg	3.640558983
AK014330	107435	Hat1	3.648040833
NM_008816	18613	Pecam1	3.660811183
BC003329	54484	Mkrn1	3.66147285
AK011596	22042	Tfrc	3.664959917
BB128741	319801	9630033F20R	3.701341
AF037370	12865	Cox7a1	3.709307883
BC006646	12224	Klf5	3.738650033
AV024881	52609	Cbx7	3.776539367
BB234337	101602	AI467606	3.805827767
AK013117	76974	1190003J15Ri	3.806894467
NM_010068	13436	Dnmt3b	3.83760305
NM_009886	12614	Celsr1	3.862550233
BE648070	58180	Hic2	3.868284683
NM_007469	11812	Apoc1	3.873187833
BC018383	226180	Ina	3.88687835
AB072269	13511	Dsg2	3.8942605
NM_013512	13824	Epb4.1l4a	3.902842033
BB473446	239157	Pnma2	3.946305617
AK010391	71988	Esco2	3.965438167
BC020144	13511	Dsg2	3.9713275

AK009960	69697	2310057J16Ri	3.9715563
AF285580	77485	Stk31	3.971668
NM_007397	11481	Acvr2b	3.974705033
BC003778	21420	Tcfap2c	4.010026117
NM_011934	26380	Esrrb	4.057861333
NM_016907	20732	Spint1	4.058743833
BC004654	19725	Rfx2	4.076663867
AV060866	18778	Pla2g1b	4.077706683
BE133749	54484	Mkrn1	4.082165
AF425084	97848	Serpinb6c	4.1037625
AV213552	24074	Taf7	4.123073
BQ175337	72685	Dnajc6	4.13664
AF104416	11828	Aqp3	4.179715783
BM210256	14106	Foxh1	4.211211117
NM_023844	67374	Jam2	4.2200985
AK019319	11816	Apoe	4.313206417
NM_031383	83557	Lin28	4.338394817
BG084230	66824	Pycard	4.33866605
AK010826	67374	Jam2	4.358034267
AF330212	12310	Calca	4.358796767
BQ173923	12808	Cobl	4.384203133
M18775	17762	Mapt	4.42236835
BC027285	68713	Ifitm1	4.4224295
AK021186	77485	Stk31	4.436390117
NM_009903	12740	Cldn4	4.437383
NM_009749	12069	Bex2	4.45785005
NM_009434	22113	Phlda2	4.474681333
BC017609	18424	Otx2	4.492426433
BC023498	72027	Slc39a4	4.528567183
AA215046	67080	1700019D03R	4.535403217
BC004037	22256	Ung	4.580378817
NM_007515	11989	Slc7a3	4.595658
BB469300	243574	Kbtbd8	4.605572983
NM_016754	17907	Mylpf	4.613079
AV277440	18020	Nfatc2ip	4.6769755
BI556771	76974	1190003J15Ri	4.717042917
BB559861	60510	Syt9	4.853818083
BB218107	16792	Laptm5	4.885893
NM_030676	26424	Nr5a2	5.081557
NM_008108	14562	Gdf3	5.14758275
NM_133982	102614	Rpp25	5.190646483
BG245669	14275	Folr1	5.20975025
NM_008652	17865	Mybl2	5.210572917
AV095095	17865	Mybl2	5.318561367
NM_009482	22286	Utf1	5.421930317
AK006110	67080	1700019D03R	5.43684025
C79957	13511	Dsg2	5.656151683

BB739342	58198	Sall1	5.673027467
BB414515	20527	Slc2a3	5.699811883
BG092030	13511	Dsg2	6.010094817
BG064756	99377	Sall4	6.034042167
BC006054	103551	E130012A19R	6.06322625
AF220524	54427	Dnmt3l	6.105105033
NM_009477	22271	Upp1	6.128136833
BB067210	73693	Dppa4	6.187330683
M75135	20527	Slc2a3	6.2282474
X69698	20527	Slc2a3	6.23042505
AV029604	22773	Zic3	6.296894567
NM_021480	58865	Tdh	6.529936483
AF176530	50764	Fbxo15	6.66547415
AK005720	71827	Lrrc34	6.691872117
BB550860	21420	Tcfap2c	6.771666083
AK010400	406217	Bex4	6.816883167
AV212609	353283	Eras	6.834029183
AV298358	381269	Mreg	6.872264583
NM_009337	21432	Tcl1	6.879203683
BB732077	22773	Zic3	6.953696833
AV099404	245128	AU018091	7.054458833
U31967	20674	Sox2	7.296394917
BB709552	14175	Fgf4	7.45980965
AV333667	26380	Esrrb	7.469081817
NM_007430	11614	Nr0b1	7.59146485
BB041571	381591	L1td1	7.663746183
NM_009556	22702	Zfp42	7.9192687
NM_009864	12550	Cdh1	7.9907572
NM_013633	18999	Pou5f1	8.090839917
AK010332	71950	Nanog	8.503540967
AV294613	21667	Tdgf1	8.80162735

Table S4 Top 20 important miRNAs in stem cells

mmu-miR-290
mmu-miR-293
mmu-miR-291a-3p
mmu-miR-292-5p
mmu-miR-295
mmu-miR-294
mmu-miR-291a-5p
mmu-miR-291b-5p
mmu-miR-302b
mmu-miR-124a
mmu-miR-182
mmu-miR-200a
mmu-miR-20b
mmu-miR-302d
mmu-miR-106a
mmu-miR-183
mmu-miR-135b
mmu-miR-690
mmu-miR-142-3p
mmu-miR-302
